# Erratum: CORRIGENDUM: Fault-tolerant quantum computation with a soft-decision decoder for error correction and detection by teleportation

**DOI:** 10.1038/srep46958

**Published:** 2018-03-23

**Authors:** Hayato Goto, Hironori Uchikawa

Scientific Reports 5: Article number: 5771; 10.1038/srep05771 published online: 06 20 2013; updated: 03 23 2018.

The original version of this Article contained a typographical error in the volume number ‘5’ was incorrectly given as ‘4’. This error has now been corrected in the PDF and HTML versions of the Article.

